# Downregulation of c-SRC kinase CSK promotes castration resistant prostate cancer and pinpoints a novel disease subclass

**DOI:** 10.18632/oncotarget.4279

**Published:** 2015-06-18

**Authors:** Chih-Cheng Yang, Ladan Fazli, Salvatore Loguercio, Irina Zharkikh, Pedro Aza-Blanc, Martin E. Gleave, Dieter A. Wolf

**Affiliations:** ^1^ Tumor Initiation & Maintenance Program, Sanford-Burnham Medical Research Institute, La Jolla, CA 92037, USA; ^2^ Functional Genomics Core, Sanford-Burnham Medical Research Institute, La Jolla, CA 92037, USA; ^3^ San Diego Center for Systems Biology, La Jolla, CA 92037, USA; ^4^ Tumor Analysis Core, Sanford-Burnham Medical Research Institute, La Jolla, CA 92037, USA; ^5^ School of Pharmaceutical Sciences & Center for Stress Signaling Networks, Xiamen University, Xiamen 361102, China; ^6^ Vancouver Prostate Centre, Vancouver, BC, Canada V6H 3Z6; ^7^ Department of Molecular and Experimental Medicine, The Scripps Research Institute, La Jolla, CA 92037, USA

**Keywords:** castration-resistant prostate cancer, c-SRC kinase CSK, mouse xenografts, human prostate cancer tissue samples, siRNA screen

## Abstract

SRC kinase is activated in castration resistant prostate cancer (CRPC), phosphorylates the androgen receptor (AR), and causes its ligand-independent activation as a transcription factor. However, activating SRC mutations are exceedingly rare in human tumors, and mechanisms of ectopic SRC activation therefore remain largely unknown. Performing a functional genomics screen, we found that downregulation of SRC inhibitory kinase CSK is sufficient to overcome growth arrest induced by depriving human prostate cancer cells of androgen. CSK knockdown led to ectopic SRC activation, increased AR signaling, and resistance to anti-androgens. Consistent with the *in vitro* observations, stable knockdown of CSK conferred castration resistance in mouse xenograft models, while sensitivity to the tyrosine kinase inhibitor dasatinib was retained. Finally, CSK was found downregulated in a distinct subset of CRPCs marked by AR amplification and ETS2 deletion but lacking PTEN and RB1 mutations. These results identify CSK downregulation as a principal driver of SRC activation and castration resistance and validate SRC as a drug target in a molecularly defined subclass of CRPCs.

## INTRODUCTION

While the bulk of prostate cancers initially depend on androgen, progression to castration resistant prostate cancer (CRPC) under androgen deprivation therapy is inevitable. The obligatory progression to castration resistance (CR) is one of the most significant challenges in the management of advanced disease. Once CR has occurred, the cancer has typically metastasized, and no life extending treatment options are currently available. The molecular pathways leading to CR remain ill defined, however, thus hampering the development of effective drugs for advanced prostate cancer.

Whereas androgen signaling maintains the differentiation and inhibits the proliferation of normal luminal prostate epithelial cells [1, 2], it is rewired in cancer cells such as to stimulate their proliferation and survival in a cell autonomous fashion [3]. Castration therefore initially inhibits the growth and induces apoptosis of tumor cells; prolonged androgen withdrawal, however, will lead to CR. This transition is typically marked by a gain of function in androgen receptor (AR) signaling [4], although the transcriptional read-out of such gain may be different from that elicited by AR signaling in hormone-sensitive prostate cancer cells (Wang et al., 2009). It has nevertheless become clear that most CRPCs express high levels of the androgen receptor (AR) and are, in fact, addicted to the AR protein even in the absence of ligand [5]. Mechanisms of ectopic AR activation identified in CRPC include AR gene amplification (~30%), mutations in the AR ligand-binding domain conferring promiscuity for other ligands, increased co-activator or reduced co-repressor recruitment (~15%), upregulation of AR mRNA, and upregulation of intra-tumoral testosterone synthesis [4–7].

In addition, several growth factor and kinase signaling pathways impinge on the AR leading to its ectopic activation [7]. For example, SRC kinase whose activity is upregulated in a significant fraction of CRPCs [8–10], interacts with the AR and directly phosphorylates and activates it in a ligand independent manner [9, 11]. SRC also potentiates AR transactivation [12] and synergizes with the AR in a reconstituted prostate carcinogenesis mouse model [13]. Conversely, depletion of SRC curtails the growth of prostate cancer xenografts in castrated mice [9]. A transcriptomic analysis has indicated SRC-AR synergism also in human CRPC [14]. Since activating SRC mutations are rare in human tumors [15, 16], increased SRC activity might arise from paracrine and/or autocrine activation of growth factor receptors [3, 9, 17] or from yet unidentified mechanisms. Significantly, a functional understanding of these kinase pathways has validated some of them as novel targets for rational intervention in CRPC. For example, the tyrosine kinase inhibitor dasatinib has undergone clinical testing in this disease [18–20].

Systematic attempts of identifying pathways contributing to CR have employed large scale profiling methods such as transcriptomics, immunoblotting, and immunohistochemistry to pinpoint expression changes in advanced prostate cancer [21]. More recently, genome sequencing has begun to reveal the genomic heterogeneity of human prostate cancer with several recurrent gene mutations and large scale rearrangements [22–24]. Exome sequencing has begun to identify genomic alterations specific to CRPC [25, 26]. While highly valuable, these genomic approaches are complicated by the uncertainty whether the observed changes are cause or consequence of the CR phenotype. Likewise, these techniques cannot assess differences in protein (enzyme) activity; for example, those that may arise from post-translational modifications. Finally, such profiling data may be skewed due to varying stromal-epithelial ratios of the samples examined. Such distinctions are essential, however, for exploiting CR pathways for the development of novel therapeutic modalities.

Causal relationships can readily be inferred de novo by loss-of-function genomic screening, but thorough validation is required [27]. Since CR is thought of as a cell autonomous phenotype of prostate cancer cells [3], we have developed a functional genomics screen in human prostate cancer cells to pinpoint signaling pathways implicated in the development of CR. Utilizing this unbiased platform, we have pinpointed downregulation of the SRC inhibitory kinase CSK as a promoter of castration resistance, validated SRC kinase as a target in CRPC, and identified a distinct subclass of human CRPCs marked by low levels of CSK that may be responsive to SRC inhibition.

## RESULTS

### Functional genomics screen implicates CSK in castration resistance

Considering the cell autonomous nature of the castration resistant phenotype [3, 4], we used the androgen responsive prostate cancer cell line LNCaP [28] to develop a loss-of-function screen to delineate signaling pathways promoting androgen-independent proliferation. Cells were switched to androgen-depleted media to arrest proliferation, and transfected with a library of siRNAs targeting 704 kinases and kinase regulators (the “kinome”), followed by determination of cell proliferation (Supplementary Figure S1A). The synthetic androgen R1881 was used as a positive control for stimulation of cell proliferation, while non-targeting siRNA was used as a negative control. Duplicate screens rendered 31 statistically significant hits. In a network analysis based on the STRING 9.1 interactome and a price collecting Steiner Forest algorithm, 28 of these hits clustered into an optimal network that featured the AR as the central node (Supplementary Figure S1B). Considering the preeminent pro-oncogenic role of the AR in CRPC [4], the network analysis provided orthogonal affirmation of the biological and medical relevance of the screen.

**Figure 1:**
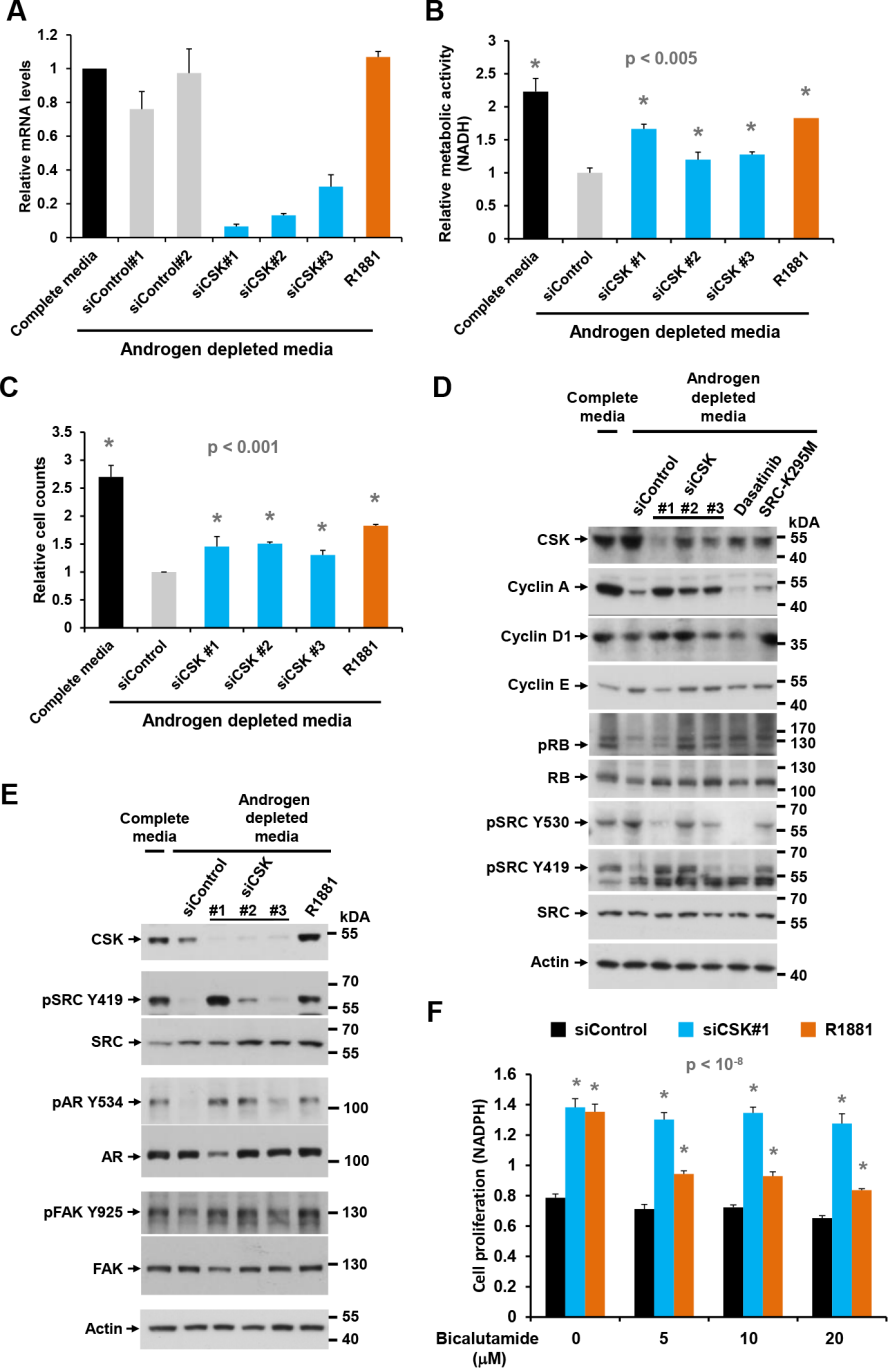
Effect of CSK knockdown on androgen-independent proliferation and SRC activity in LNCaP cells **A.** LNCaP cells were depleted of androgen for 72 h, followed by transfection of different siRNAs targeting CSK. After 48 h, cells were harvested for determination of knockdown efficiency by Q-PCR. **B, C.** Cell proliferation was determined with the MTS assay and by cell counting. The synthetic androgen R1881 was used as positive control. Error bars represent standard deviations of 3 – 6 replicate measurements. Significance of the differences to the siControl sample (grey bar) was assessed by calculating *p* values using an unpaired *t*-test, two-tailed distribution, assuming equal variance. Asterisks denote significant differences. **D, E.** Protein lysates obtained under the same conditions were probed with antibodies for cell cycle markers and markers of SRC activity. Dasatinib treatment and overexpression of the dominant negative SRC-K295M mutant was used to confirm the identity of the pSRC Y419 and Y530 bands, both of which were eliminated by the tyrosine kinase inhibitor. **F.** The anti-androgen bicalutamide was added to cells at the time of siRNA transfection to determine the dependence of cell proliferation induced by CSN knockdown on AR signaling. Error bars represent standard deviations of 7 replicate measurements. Significance of the differences to the androgen depleted sample (black bar) was assessed by calculating *p* values using an unpaired *t*-test, two-tailed distribution, assuming equal variance. Asterisks denote significant differences relative to the siControl values (black bars).

One of the top ranking screening hits was C-terminal SRC kinase (CSK, Supplementary Figure S1C), an enzyme that inhibits SRC family kinases (SFKs) by phosphorylating a C-terminal tyrosine (Y530 in human SRC [29]). Knockdown of CSK in androgen depleted LNCaP cells with three distinct siRNAs of varying efficiencies (Figure 1A, 1D, 1E) led to increases in cell proliferation, cell number, and the fraction of cells in S phase, similar in extent to what was achieved with the positive control R1881 (Figure 1B, 1C; Supplementary Figure S1D). Cell cycle entry mediated by CSK knockdown coincided with increased levels of cyclin A, cyclin D1 and RB phosphorylation, whereas cyclin E levels remained largely unchanged (Figure 1D). Consistent with increased SRC activity, the inhibitory phosphorylation on Y530 was reduced whereas the activating phosphorylation on Y419 was increased upon knockdown of CSK (Figure 1D, 1E). Treatment of cells with the tyrosine kinase inhibitor dasatinib and overexpression of the dominant negative SRC mutant SRC-K295M were used to authenticate the Y530 and Y419 species (Figure 1D).

Elevated SRC activity was also indicated by tyrosine phosphorylation of known downstream targets such as focal adhesion kinase (FAK, Y925; [30]) and the AR (Y534; Figure 1E). Notably, SRC-mediated phosphorylation of the AR on Y534 was previously shown to confer ligand-independent AR activation [9]. In addition, knockdown of CSK increased PSA secretion (Supplementary Figure S1D), further suggesting ectopic AR activation via SRC. Whereas cell proliferation induced by R1881 was inhibited by the AR blocker bicalutamide, proliferation mediated by CSK knockdown was not (Figure 1F). This observation suggests that ectopic AR activation in response to CSK knockdown occurs in a ligand-independent fashion. The above effects were most pronounced with CSK siRNA #1, which showed the strongest and most consistent activity in suppressing CSK levels (Figure 1A, 1D, 1E). This apparent dose dependence suggests a specific on-target effect of the relevant siRNA [27].

**Figure 2:**
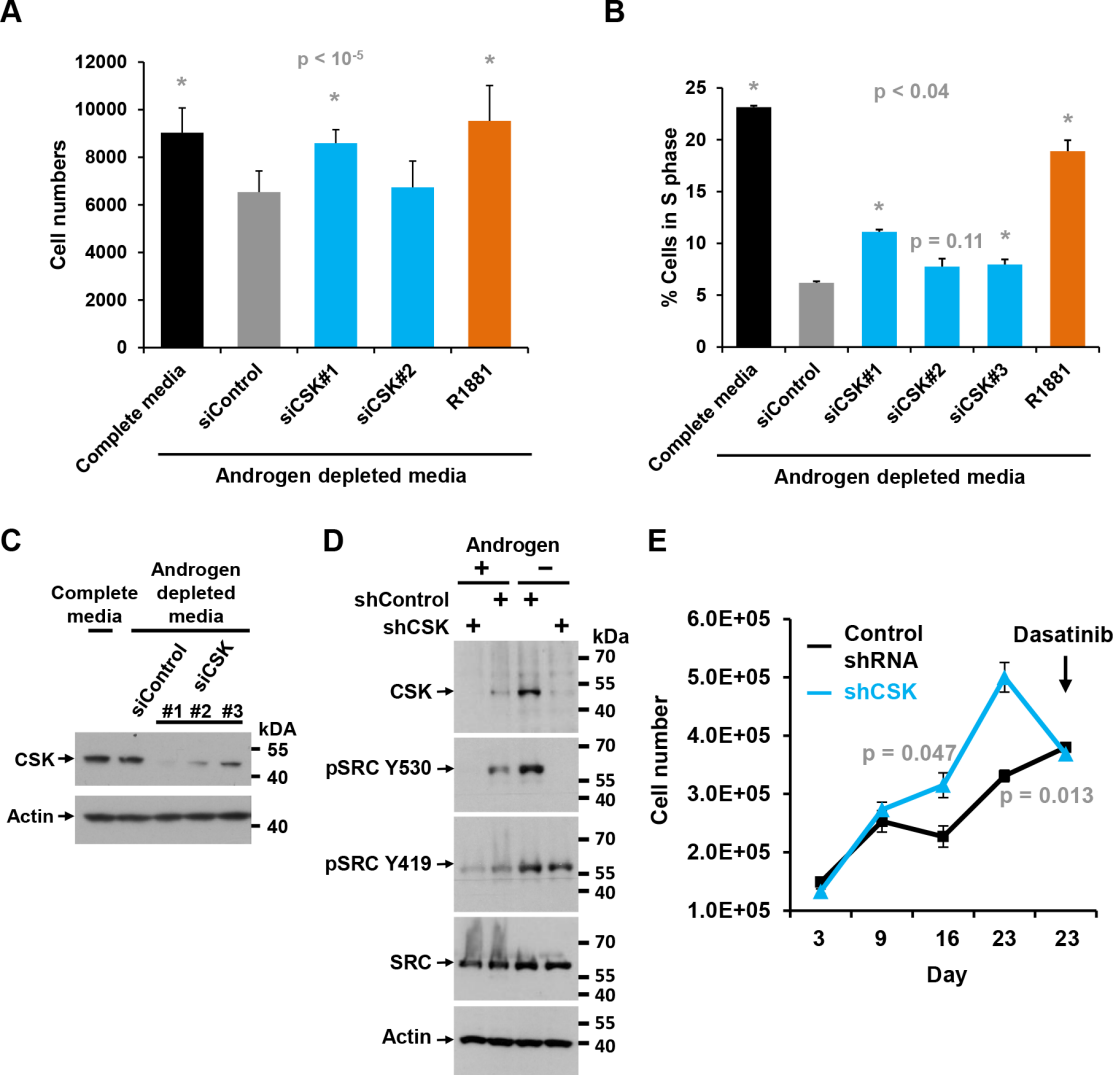
Effect of CSK knockdown on androgen-independent proliferation and SRC activity in LAPC4 cells **A.** LAPC4 cells were maintained in androgen-depleted media, followed by knockdown of CSK or re-addition of androgen (R1881). Cell numbers were determined. Error bars represent standard deviations of 12 replicate measurements. Significance of the differences to the siControl sample (grey bar) was assessed by calculating *p* values using an unpaired *t*-test, two-tailed distribution, assuming equal variance. Asterisks denote significant differences. **B.** Same experiment as in (A) but the fraction of cells in S phase was determined by flow cytometry. Error bars represent standard deviations of 2 replicate measurements. Significance of the differences to the siControl sample (grey bar) was assessed by calculating *p* values using an unpaired *t*-test, two-tailed distribution, assuming equal variance. Asterisks denote significant differences. **C.** Immunoblot to document the effect of various siRNAs targeting CSK upon transfection into LAPC4 cells. The signal obtained for actin is shown as a reference. **D.** Immunoblot to demonstrate efficient knockdown of CSK upon infection of LAPC4 cells with lentiviruses driving the expression of shRNA directed against CSK relative to Control shRNA. Blots were also probed with a marker of SRC activity (SRC Y530). Actin is shown as a reference. **E.** The LAPC4 cells with stable shRNA-mediated knockdown of CSK shown in (C) were grown in media containing charcoal-stripped and thus androgen-deprived FBS, and cell numbers were determined at the indicated times (Error bars represent standard deviations of 2 replicates). Dasatinib was added at the time indicated, demonstrating that androgen-independent proliferation promoted by CSK knockdown is reversed by the tyrosine kinase inhibitor. Significance of the differences between the shControl and shCSK samples was assessed by calculating *p* values using an unpaired *t*-test, two-tailed distribution, assuming equal variance. Time points showing significant differences between shCOntrol and shCSK cells are highlighted by stating the respective *p* values.

The effects of CSK knockdown on androgen-independent proliferation were corroborated in another androgen responsive prostate cancer cell line, LAPC4. As in LNCaP cells, CSK siRNA #1 led to an increase in cell numbers and the fraction of cells in S phase under androgen deprived conditions (Figure 2A, 2B). In addition, stable knockdown of CSK by lentiviral transduction of shRNA led to downregulation of CSK and activation of SRC as indicated by a decrease in the inhibitory phosphorylation on Y530 (Figure 2C). Stable knockdown of CSK also allowed LAPC4 cells to proliferate more efficiently in androgen depleted media than control knockdown cells (Figure 2D). Importantly, androgen-independent proliferation was still inhibited by dasatinib (Figure 2D).

**Figure 3:**
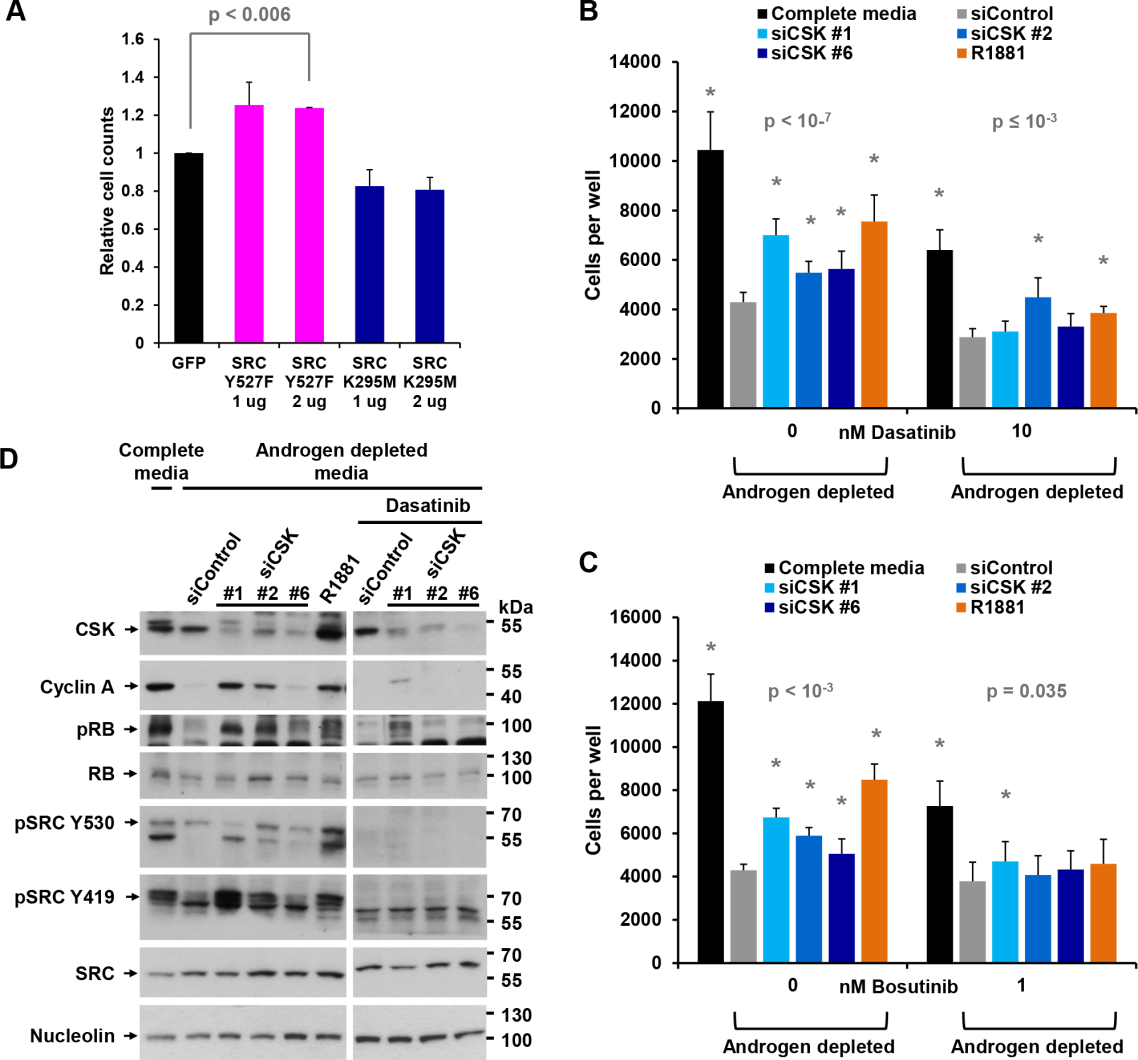
Androgen-independent growth induced by CSK knockdown is mediated by SRC activity **A.** LNCaP cells were androgen-depleted for 72 h, followed by transfection with plasmids driving the expression of constitutively active SRC-Y527F or dominant negative SRC-K295M. Cells numbers were counted after 72 h. Expression of GFP was used as a negative control. Error bars represent standard deviations of 2 replicates. Significance of the differences to the GFP transfected control sample (black bar) was assessed by calculating *p* values using an unpaired *t*-test, two-tailed distribution, assuming equal variance. Asterisks denote significant differences. **B, C.** The indicated doses of the tyrosine kinase inhibitors dasatinib or bosutinib were added at the time of CSK knockdown and cell numbers were determined. Error bars represent standard deviations from 12 - 16 measurements. Significance of the differences to the siControl sample (grey bar) was assessed by calculating *p* values using an unpaired *t*-test, two-tailed distribution, assuming equal variance. Asterisks denote significant differences. **D.** CSK knockdown cells were treated with dasatinib, and the indicated markers were assayed by immunoblotting.

Like CSK knockdown, transfection of LNCaP cells with constitutively active SRC-Y527F (where the CSK target tyrosine is mutated and therefore cannot be phosphorylated), but not with dominant negative SRC-K295M, was able to promote the proliferation of androgen-depleted LNCaP cells (Figure 3A). Of note, dominant negative SRC-K295M was unable to augment downregulation of cyclin A expression and RB phosphorylation beyond the level obtained by androgen depletion, suggesting that hormone withdrawal led to maximal SRC suppression (Figure 1D). In contrast, the tyrosine kinase inhibitors dasatinib and bosutinib largely prevented cell proliferation induced by CSK knockdown and R1881 administration (Figure 3B, 3C). Dasatinib also prevented SRC phosphorylation on Y419 and upregulation of cyclin A levels and RB phosphorylation as a consequence of CSK knockdown (Figure 3D). These data firmly suggest that androgen-independent proliferation of LNCaP cells induced by knockdown of CSK is mediated through increased SRC-mediated AR activation.

### Stable knockdown of CSK confers castration resistance in prostate cancer xenograft models

**Figure 4:**
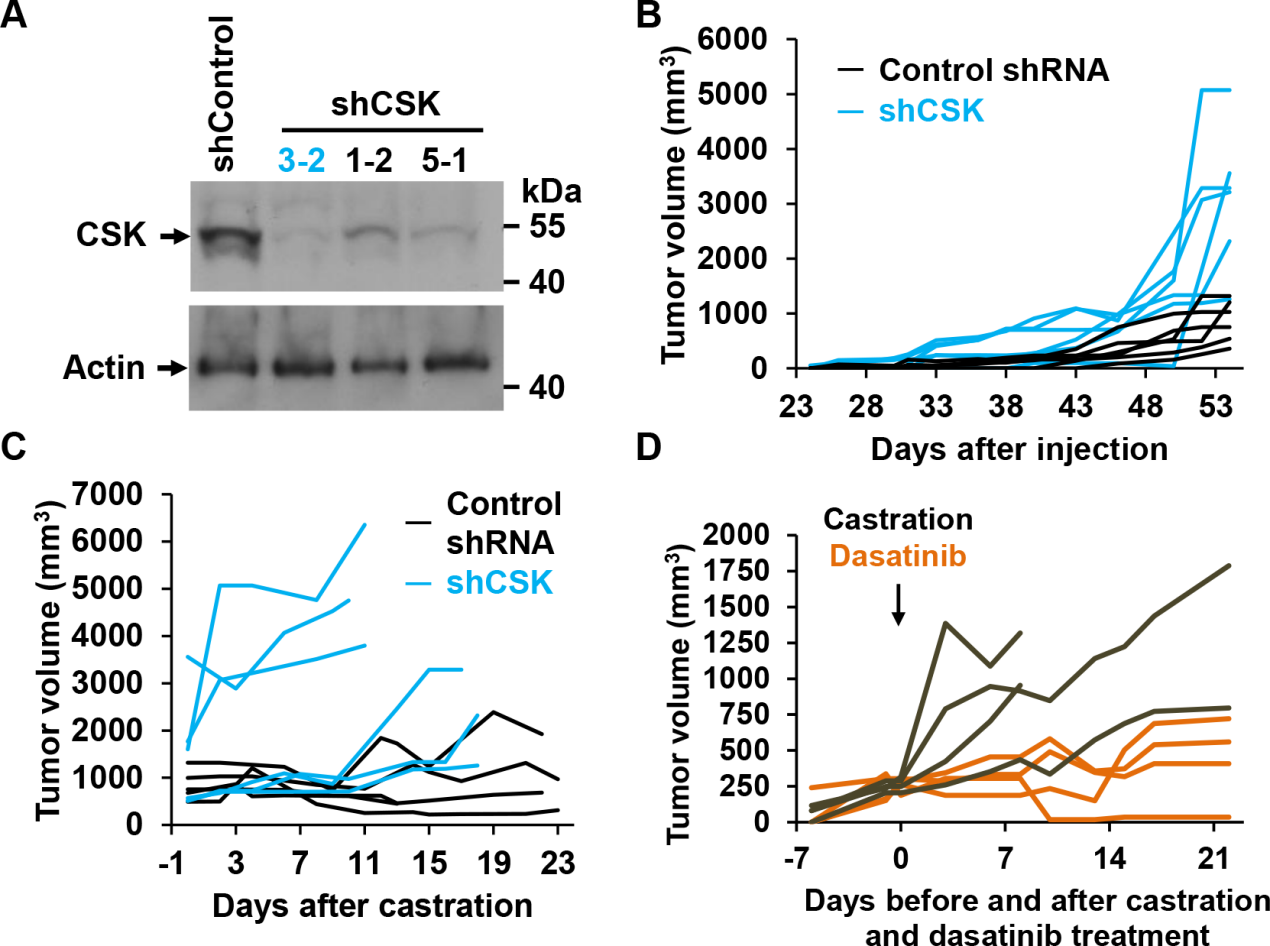
CSK knockdown confers castration resistance *in vivo* **A.** Immunoblot to demonstrate efficient knockdown of CSK upon infection of LNCaP cells with lentiviruses driving the expression of CSK relative to Control shRNA. **B.** shCSK knockdown LNCaP cells (clone 3-2) were injected into SCID mice, and tumor growth was monitored for the indicated periods of time. Each line represents tumor sizes in a single animal over time. **C.** Mice were castrated at the indicated tumor volumes and tumor growth was followed for the indicated periods. Each line represents tumor sizes in a single animal over time. **D.** An independent set of mice was injected with shCSK3-2 cells, followed by castration when tumors had reached a volume of ~200–300 mm^3^. At the time of castration, tumor bearing mice were dosed with dasatinib (50 mg/ml p.o. daily) for the indicated periods, and tumor growth was monitored. Dasatinib suppressed the growth of LNCaP cells rendered castration resistant through knockdown of CSK. Each line represents tumor sizes in a single animal over time.

To test the effect of CSK downregulation in CRPC *in vivo*, we prepared LNCaP cell lines in which CSK was stably downregulated through either one of three lentivirally delivered shRNAs. Cells from clone 3-2, which exhibited the most efficient downregulation of CSK (Figure 4A) failed to arrest proliferation upon androgen depletion and were only minimally stimulated by R1881 (Supplementary Figure S2). When CSK knockdown cells were injected subcutaneously into male SCID mice, tumors formed from CSK knockdown cells (shCSK) appeared earlier and grew to larger sizes than those obtained with control knockdown cells (shControl; Figure 4B). Upon castration, shControl tumors either regressed or stopped growing, whereas shCSK LNCaP tumors continued to increase in size (Figure 4C). Likewise, tumors derived from LAPC4 cells in which CSK was stably knocked down (Figure 2C) grew in a castration resistant manner (Supplementary Figure S3A). These tumors displayed low levels of CSK and correspondingly low levels of pSRC Y530 (Supplementary Figure S3B). When castrated mice bearing castration resistant shCSK LNCaP tumors were dosed with dasatinib (50 mg/kg p. o., once daily), tumor growth was suppressed relative to mock treated animals (Figure 4D). Taken together, these findings demonstrate that downregulation of CSK is sufficient to confer a castration resistant phenotype to androgen responsive prostate cancer cells *in vivo*. At the same time, regimens directed toward inhibiting SRC activity appear efficacious in the treatment of CRCP.

### Low CSK defines a subclass of human CRPCs

**Figure 5:**
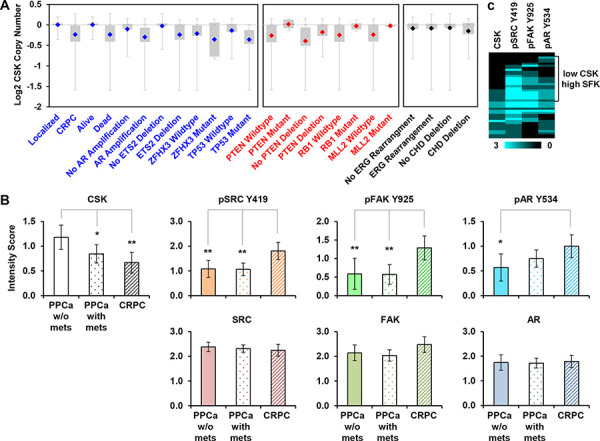
CSK expression in human CRPC **A.** CSK gene copy number data from Grasso et al. (Ref. [25]) were drawn from the Oncomine database. Shown are events that are positively (blue) or negatively (red) correlated with loss of CSK. Uncorrelated events are shown in black type. **B.** The indicated markers were quantified in a panel of prostate cancer tissues by immunohistochemistry. Scoring was on a scale from 0 to 3 (see Materials and Methods). Error bars represent 2 standard errors which correspond approximately to 95% confidence intervals (CI). Significance was estimated by assessing the extent of overlap of the CI bars, considering that for *n* ≥ 10, an overlap of 0.5 indicates a *p* value of ~0.05, whereas an overlap of 0 corresponds to a *p* value of ~0.01 [40]. In the experiment shown, n in each group = 25 – 52 cores, see Table S1. Thus, * denotes *p* ≈ 0.05, **p ≈ 0.01. PPCa = primary prostate cancer; mets = metastases. **C.** The scores of 42 informative CRPC cores were clustered (similarity metric: absolute correlation uncentered, clustering method: single linkage), and visualized as an intensity map. SFK = SRC Family Kinase.

While an increase in SRC activity has been reported in CRPC, it remained unclear how this might occur [10]. Since mutational activation of SRC is exceedingly rare [15, 16], we considered the possibility that CSK might be downregulated in a subset of CRPCs. A search in the Oncomine database revealed frequent CSK copy number losses specifically in CRPCs as compared to primary prostate cancer (Figure 5A; data from [25]). A similar observation was made with an independent dataset (Supplementary Figure S4A; [31]) as well as for the CSK-related tyrosine kinase MATK (Supplementary Figure S4B). CSK copy number loss was correlated with poor survival, AR amplification, and deletion/mutation of several tumor suppressors (ETS2, ZFHX3, and TP53; [25]). Negative correlations were seen with mutational inactivation of other regulators previously implicated in CRPC such as PTEN and RB1 [32–34]. No correlation with ERG rearrangements or deletion of CDH was apparent (Figure 5A), suggesting that CRPCs with CSK copy number loss may constitute a distinct subclass.

We next performed immunohistochemistry on a panel of prostate cancer tissue samples representing different disease stages (localized, metastatic, castration resistant). Using siRNA-mediated knockdown of CSK in LNCaP cells that were subsequently embedded in paraffin, we first established the specificity of a commercially available CSK antibody in immunohistochemistry (Supplementary Figure S5A). Staining of the prostate cancer progression panel with this validated antibody revealed a significant decrease in average CSK reactivity in metastatic prostate cancer and CRPCs relative to primary prostate cancers (Figure 5B, Supplementary Figure S5B). As reported previously [9, 10], pSRC Y419 and pAR Y534 were higher in CRPC than in primary prostate cancers (Figure 5B, Supplementary Figure S5B). The same was true for another SRC target, pFAK Y925. Total SRC, AR, and FAK levels were not significantly changed in the progression series (Figure 5B). Comparative analysis of individual CRPC cores revealed an apparent subclass of tumors marked by low levels of CSK and high activity of SFKs as determined by pSRC Y419, pFAK Y925, and pAR Y534 intensity (Figure 5C). These data suggest that CSK protein expression is downregulated in a sizeable subclass of CRPCs (~50% of CRPC cores) thus resulting in increased SFK activity.

## DISCUSSION

The data obtained in our unbiased loss-of-function screen indicate CSK downregulation as a prominent driver of progression to castration resistance. Castration resistance of androgen sensitive LNCaP and LAPC4 cells in response to CSK knockdown is likely mediated through activation of SRC or a SRC family kinase, because it is abolished by tyrosine kinase inhibitors dasatinib and bosutinib *in vitro* and *in vivo*. Our immunohistochemical studies also indicate downregulation of CSK as a predominant mechanism of SRC activation in a significant fraction of CRPCs. Together, the results suggest CSK as a tumor suppressor, a role that has previously been inferred from the observation of CSK downregulation in hepatocellular carcinoma [35]. Since overexpression of CSK inhibits SRC-mediated AR tyrosine phosphorylation [11], ectopic AR phosphorylation and activation, as indicated by increased PSA secretion, is the most likely mechanism driving castration resistance in response to CSK downregulation.

The mechanisms of CSK downregulation at the transition to CRPC remain unknown, but our mining of published datasets implicates allelic loss while not excluding complimentary events such as promoter methylation, miR overexpression, and increased proteolytic turn-over of CSK protein amongst others. Regardless, our observations that (i.) knockdown of CSK abolishes the growth inhibitory effects of the androgen receptor blocker bicalutamide, and (ii.) CSK protein is upregulated in response to androgen withdrawal in LNCaP and LAPC4 cells (Figure 1D, 2C) strongly suggest that commonly administered androgen deprivation therapy may impose selective pressure on tumor cells to downregulate CSK levels/activity for progression to castration resistance.

Using an entirely unbiased genetic loss-of-function strategy, our findings have reinforced the preclinical evidence suggesting SRC as a valid drug target in CRPC [8–10, 13, 14]. This concept gained initial momentum by encouraging results in multiple phase II clinical trials with the tyrosine kinase inhibitor dasatinib [18–20]. Nevertheless, dasatinib, a drug that successfully controlled castration-resistant shCSK knockdown tumors in our xenograft experiments, has disappointed in a recently concluded phase III trial in chemotherapy naïve patients. The primary endpoint was overall survival of CRPC patients treated with a combination of dasatinib and docetaxel relative to docetaxel alone [36]. The rationale for combining dasatinib with docetaxel has subsequently been questioned noting the limited preclinical evidence supporting additive or synergistic effects [37]. An added limitation of the failed Phase III trial was that it neither assessed the status of SRC activity in enrolled CRPC patients nor the effect of dasatinib on SRC activity upon treatment.

Our results support a renewed interest in the potential of SRC inhibitors in CRPC by suggesting CSK downregulation as a novel marker for sensitivity of CRPCs to dasatinib or more advanced SRC inhibitors. Whereas SRC activation may not be a rate-limiting factor in all CRPCs, it may be a critical bottleneck in CRPCs displaying CSK downregulation as suggested by our xenograft studies. Secondly, our data suggest pathways for intervention in combination with SRC inhibitors for which there exists a stronger rationale than for docetaxel. For example, loss of CSK appears to correlate with wildtype RB1 status. Given the critical role of RB1 inactivation in CRPC [33], tumors with low CSK may inactivate the RB1 pathway through other means such as overexpression of cyclin D or downregulation of p15 and p16 [34, 38, 39], which lead to CDK4-dependent RB1 phosphorylation and inactivation of its E2F1 repressive activity. Thus, CSK low CRPCs may be sensitive to a combination of SRC and CDK4 inhibition. Our data also suggest that CSK low CRPCs might respond to inhibition of pathways and cellular processes set into motion by loss of ETS2 and ZFHX3 function, two tumor suppressors specifically involved in the transition to CRPC [25], although regimens to do so remain to be discovered.

## MATERIALS AND METHODS

### Tissue culture, plasmids, viruses, antibodies

The human prostate cancer cell line LNCaP was obtained from ATCC and maintained in RPMI 1640 supplemented with 10% fetal bovine serum, 50 units/ml penicillin, and 50 units/ml streptomyc in (Life Technology). The SRC Y527F and K295M expression plasmids were gifts from Dr. Sara Courtneidge. The siRNAs against CSK were purchase from Ambion (Life Technologies, Grand Island, NY, #1 ID: 511; #2 ID: 513; #3 ID: s3612) or from GE Dharmacon (#6 ID: Cat# D-003110-06). The shRNAs against CSK were from Sigma-Aldrich (St. Louis, MO; #1 ID: TRCN0000000804; #3 ID: TRCN0000010009 and #5 ID: TRCN0000199500).

For DNA transfection, LNCaP cells were grown to 50−70% confluence on a 100 mm dish and transfected with 10 ug of plasmid DNA using Lipofectamin 2000 reagent according to the recommendations of the manufacturer (Life technologies, Grand Island, NY).

The following antibodies were used: CSK mouse monoclonal (Becton Dickinson, San Jose, CA), cyclin A (Clone 6E6) and cyclin E (HE12) was from Thermo Fisher (Waltham, MA). Rabbit polyclonal cyclin D1 (#2922), pRB (#9307), pSRC Y530 (#2105), pSRC Y419 (#6943), SRC (#2123), pFAK Y925 (#3284), FAK (#3285) and mouse monoclonal RB (#9309) were purchased from Cell Signaling. Mouse monoclonal AR antibody (441) was from Santa Cruz, R1881 was obtained from PerkinElmer (Waltham, MA), bicalutamide, dasatinib and busotinib were purchased from LC Laboratories (Woburn, MA)

### High-throughput RNAi screens and network analysis

A human kinome library (Ambion, Life technologies, Grand Island, NY) containing siRNAs targeting 704 kinases and kinase regulators was used for screening. Assay plates (384-well plate with optical bottom; Greiner) were spotted with 1 μl of 0.5 μM pooled siRNAs (4 siRNAs per gene in duplicate) using the Valocity 11-Bravo Pipettor with a 384 ST head. R1881 was used as positive control and included in all plates. Reverse transfection was performed using Lipofectamine RNAiMAX; final siRNA concentration was 10 nM. LNCaP cells were suspended in RPMI 1640 media maintained in 10% charcoal stripped FBS for 2 days and seeded onto assay plates using the Matrix-Well Mate (2,000 cells in 40 μl medium for each well). Cells were incubated in sealed assay plates in a 37°C incubator for four days. MTS Cell Proliferation Assay (Promega) was used for monitoring cell proliferation by adding 10 μl of MTS/PMS reagents to each well. After 2 hours incubation, reactions were stopped by adding 10 μl of 10% SDS and plates were read in a Flexstation 3 plate reader at 490 nm (Molecular Devices). For plate normalization, quantification data was converted to *Z* scores using 48 unique non-targeting siRNAs included to each plate as references: *Z* score = (*X* i – median of 48 control siRNAs)/1.4826 × MAD of 48 control siRNAs, where *X* i is quantification data and MAD is median absolute deviation. Genes were defined as primary screening hits if *Z* scores ≥ 1.8. Thirty one genes were selected for the follow-up confirmation screen (Supplementary Figure S1A).

The 31 hit genes were further studied by network analysis based on the STRING 9.1 interactome and a price collecting Steiner Forest algorithm with the screening Z scores set as node prizes and the edge weights set as the edge costs. Randomization analysis with 1000 iterations revealed a *p* value of 0.045 for enrichment of the AR in a network built from the protein interactions of 31 hit kinases relative to 31 random kinases. In addition, the AR node contained in the network identified with the 31 hit kinases had a betweenness centrality of 0.0415, which was higher than that obtained for the AR in any of the corresponding random networks.

### Confirmation screen

For the confirmation screen, 31 genes were selected from the primary screen based on their Z score (*Z* > 1.86). LNCaP cells (2,000 cells per well) were hormone-deprived and individually transfected with the siRNAs that were present in the original pool used in the primary screen. 4 additional distinct siRNAs from Dharmacon (Thermo Fisher Scientific) for these genes were also included. The confirmation screen was based on measurement of (1) the MTS proliferation assay and (2) cell counts using Celigo cytometer. Genes were defined as confirmed if at least three siRNAs showed Z scores ≥ 1.8. 12 genes were selected based on these criteria.

### Flow cytometry

Flow cytometry analysis of cell cycle distribution was performed in LNCaP cells following transfection with siRNAs. LNCaP cells (200,000 per well) were cultured in androgen deprived media for 2 days, followed by reverse transfection with 10 nM siRNAs in duplicate in 6-well plates. After 48 hours, cells were trypsinized, washed with PBS twice and then fixed with 70% ethanol overnight at −20^°^C. For FACS analysis, cells were resuspended by vortexing in 250 μl staining solution (PBS, 1% Tween 20, 10 μg/ml RNase A (Sigma), 50 μg/ml propidium iodide) and incubated for 1 hour. Samples were analyzed by flow cytometry (FACSCalibur with CellQuest software), collecting 20,000 total (ungated) events with threshold = 10 and FL2 voltage ~430 (adjusted for each sample so that 2N peak on DNA-area histogram was centered at 200). Cell cycle analysis was performed on histograms of gated counts per DNA-area (FL2-A) by the Watson (pragmatic) curve-fitting algorithm to determine the distribution of 2N, 4N and > 4N cells using FlowJo software (Tree Star, Ashland, OR).

### Cell staining and fluorescence-based assays using Celigo

To determine cell numbers after siRNA transfection in 384 well plates, 2000 cells were plated as described above, incubated for three days and nuclei were stained with Hoechst 33342. Plates were read using the adherent cell cytometer, Celigo Imaging Cell Cytometer (Brooks Life Science Systems) equipped with a brightfield and three fluorescence channels. Gating parameters were adjusted to exclude background and other non-specific signals. The total cell number in one well was equal to the total counts of gated events.

### RNA extraction and Q-RT-PCR

RNA was extracted using the RNeasy Mini kit (Qiagen). RT-PCR was performed using Power Sybr Green Mastermix (Ambion, Foster City, CA) and a Stratagene™ Mx3000p Q-RT-PCR system (Stratagene, La Jolla, CA). Primers used for detecting CSK expression were (GGCTCTACATCGTCACTGAG and CTCAGACACCAGCACATTG). GAPDH was used for internal control. Gene knockdown was calculated using the ΔΔ-Ct method.

### Generation of stable clones

The 293T producer cell line was transfected with shRNA expressing lentiviral constructs and packaging plasmid mix (System Biosciences) using Lipofectamine 2000 (Life Technologies, Grand Island, NY). shCSK lentiviral vectors (TRCN0000000804, TRCN0000010008, TRCN0000010009, TRCN0000199031, TRCN0000199500) were obtained from Sigma-Aldrich. Supernatants were collected 48 h after transfection, filtered using a 0.45-μm-pore-size nitrocellulose filter, and applied to LNCaP and LAPC4 cells. After 48 h, the cells were selected in puromycin (2 ug/ml) containing media. As a control, cells were infected with a lentivirus expressing scrambled control shRNA (Sigma-Aldrich).

### Xenograft study

8 week old male NOD/SCID mice were subcutaneously injected in one spot over the flank with a volume of 0.2 ml of prostate cancer cells (LNCaP and LAPC4 derivatives as indicated in the figures) in 50% Matrigel (BD Biosciences). Tumor growth was monitored daily using external calipers. If applicable, animals were castrated, and tumor growth was monitored for 2–3 weeks. For dasatinib treatment, animals were dosed by oral gavage daily (50 mg/kg p. o.). Mice were monitored daily for signs of distress and euthanized within 24 hours in case of distress or when tumors reached a diameter of 2 cm.

### Immunohistochemistry

This study was done on the total of 76 prostate cancer specimens obtained from the Vancouver Prostate Centre Tissue Bank. The H&E slides were reviewed and the desired areas were marked on them and their correspondent paraffin blocks. Tissue microarrays (TMAs) were manually constructed (Beecher Instruments, MD, USA) by punching duplicate cores of 1 mm diameter for the tumor specimens. All specimens were from radical prostatectomy except the 28 CRPC samples which were obtained by transurethral resection of the prostate (TURP). Supplementary Table 1 shows detailed information on the Progression Array, 2011.

Immunohistochemical staining was conducted by Ventana autostainer model Discover XT™ (Ventana Medical System, Tuscan, Arizona) with enzyme labeled biotin streptavidin system and solvent resistant DAB Map kit. The antibodies are described above. TMA slides were scanned in a Leica digital imaging system, and images were viewed using Digital Image Hub, Slide Path, Digital Pathology Solution (Dublin, Ireland). Values on a four-point scale were assigned to each sample. Descriptively, 0 represents no staining of any tumor cells, 1 represents faint or focal staining, 2 represents a strong signal in a minority of cells, and 3 represents a strong signal in the majority of cells. Staining scores were averaged across all cores of each tumor class and displayed in a bar graph with 95% confidence intervals indicated. The scores of individual CRPC cores were clustered (similarity metric: absolute correlation uncentered, clustering method: single linkage), and visualized as an intensity map.

## SUPPLEMENTARY FIGURES AND TABLE


