# Correction: Downregulation of c-SRC kinase CSK promotes castration resistant prostate cancer and pinpoints a novel disease subclass

**DOI:** 10.18632/oncotarget.28693

**Published:** 2025-02-12

**Authors:** Chih-Cheng Yang, Ladan Fazli, Salvatore Loguercio, Irina Zharkikh, Pedro Aza-Blanc, Martin E. Gleave, Dieter A. Wolf

**Affiliations:** ^1^Tumor Initiation & Maintenance Program, Sanford-Burnham Medical Research Institute, La Jolla, CA 92037, USA; ^2^Functional Genomics Core, Sanford-Burnham Medical Research Institute, La Jolla, CA 92037, USA; ^3^San Diego Center for Systems Biology, La Jolla, CA 92037, USA; ^4^Tumor Analysis Core, Sanford-Burnham Medical Research Institute, La Jolla, CA 92037, USA; ^5^School of Pharmaceutical Sciences & Center for Stress Signaling Networks, Xiamen University, Xiamen 361102, China; ^6^Vancouver Prostate Centre, Vancouver, BC, Canada V6H 3Z6; ^7^Department of Molecular and Experimental Medicine, The Scripps Research Institute, La Jolla, CA 92037, USA

**This article has been corrected:** Oncotarget has completed its investigation of this paper. Our image forensic analysis found only one issue: a duplicated western blot panel in Figure 1. The corresponding author Dr. Dieter A. Wolf confirmed that in Figure 1E, the SRC panel is an accidental duplicate of the SRC panel in Figure 3D. The authors provided original, unmodified raw data for this experiment and the date stamp confirms its authenticity. The corrected Figure 1, which was produced using the original data, is shown below. The authors declare that this correction does not affect the results or conclusions of the paper.

Original article: Oncotarget. 2015; 6:22060–22071. 22060-22071. https://doi.org/10.18632/oncotarget.4279

**Figure 1:**
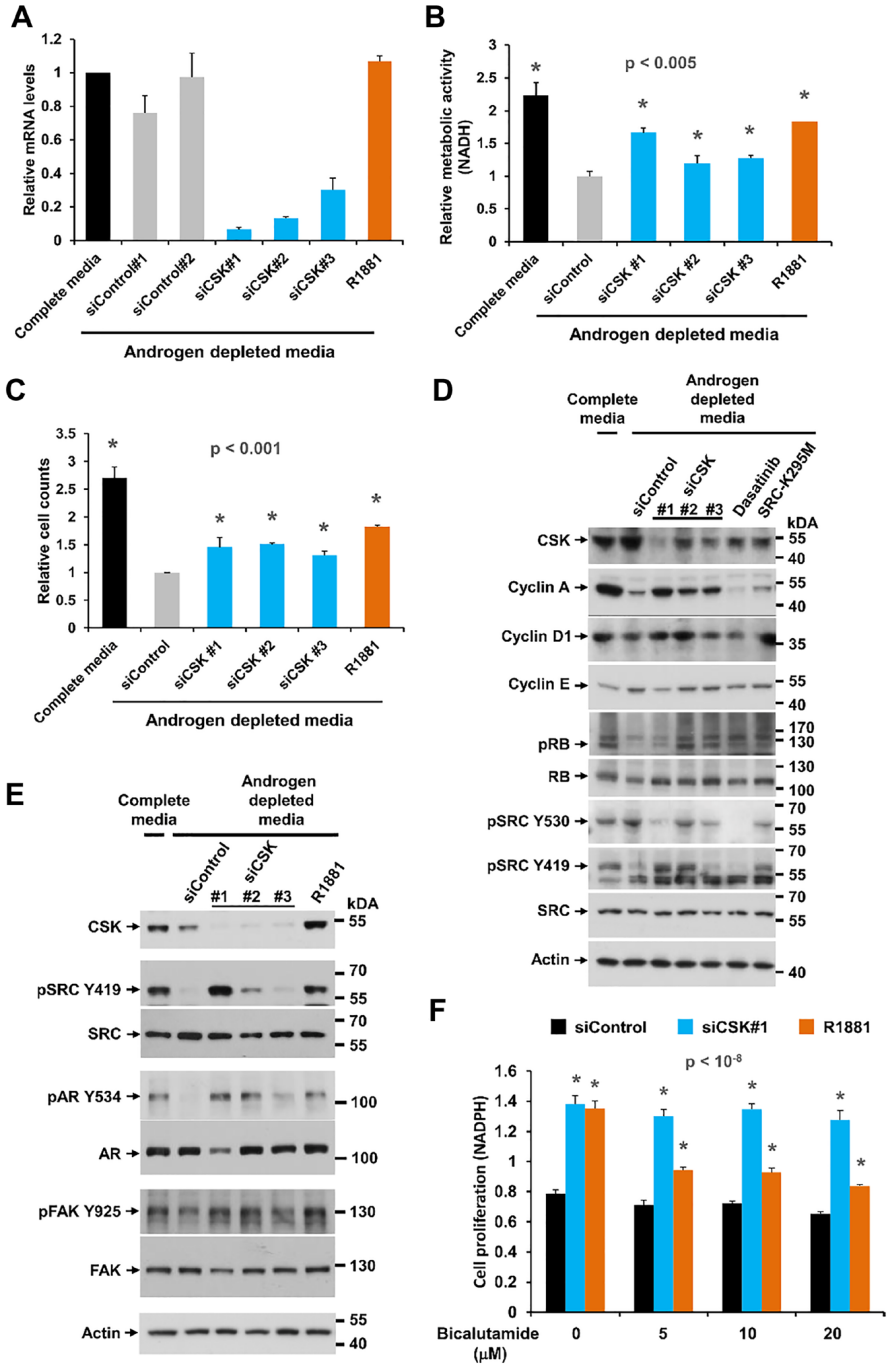
Effect of CSK knockdown on androgen-independent proliferation and SRC activity in LNCaP cells. (**A**) LNCaP cells were depleted of androgen for 72 h, followed by transfection of different siRNAs targeting CSK. After 48 h, cells were harvested for determination of knockdown efficiency by Q-PCR. (**B**, **C**) Cell proliferation was determined with the MTS assay and by cell counting. The synthetic androgen R1881 was used as positive control. Error bars represent standard deviations of 3 – 6 replicate measurements. Significance of the differences to the siControl sample (grey bar) was assessed by calculating p values using an unpaired t-test, two-tailed distribution, assuming equal variance. Asterisks denote significant differences. (**D**, **E**) Protein lysates obtained under the same conditions were probed with antibodies for cell cycle markers and markers of SRC activity. Dasatinib treatment and overexpression of the dominant negative SRC-K295M mutant was used to confirm the identity of the pSRC Y419 and Y530 bands, both of which were eliminated by the tyrosine kinase inhibitor. (**F**) The anti-androgen bicalutamide was added to cells at the time of siRNA transfection to determine the dependence of cell proliferation induced by CSN knockdown on AR signaling. Error bars represent standard deviations of 7 replicate measurements. Significance of the differences to the androgen depleted sample (black bar) was assessed by calculating *p*-values using an unpaired *t*-test, two-tailed distribution, assuming equal variance. Asterisks denote significant differences relative to the siControl values (black bars).

