# Supplementary material for: Downregulation of c-SRC kinase CSK promotes castration resistant prostate cancer and pinpoints a novel disease subclass
Source: Oncotarget. 2015 Jun 18;6(26):22060–71. doi: 10.18632/oncotarget.4279 (PMC4673146; doi:10.18632/oncotarget.4279)
Supplement: Supplementary file 1 [file oncotarget-06-22060-s001.pdf]

SUPPLEMENTARY FIGURES AND TABLE

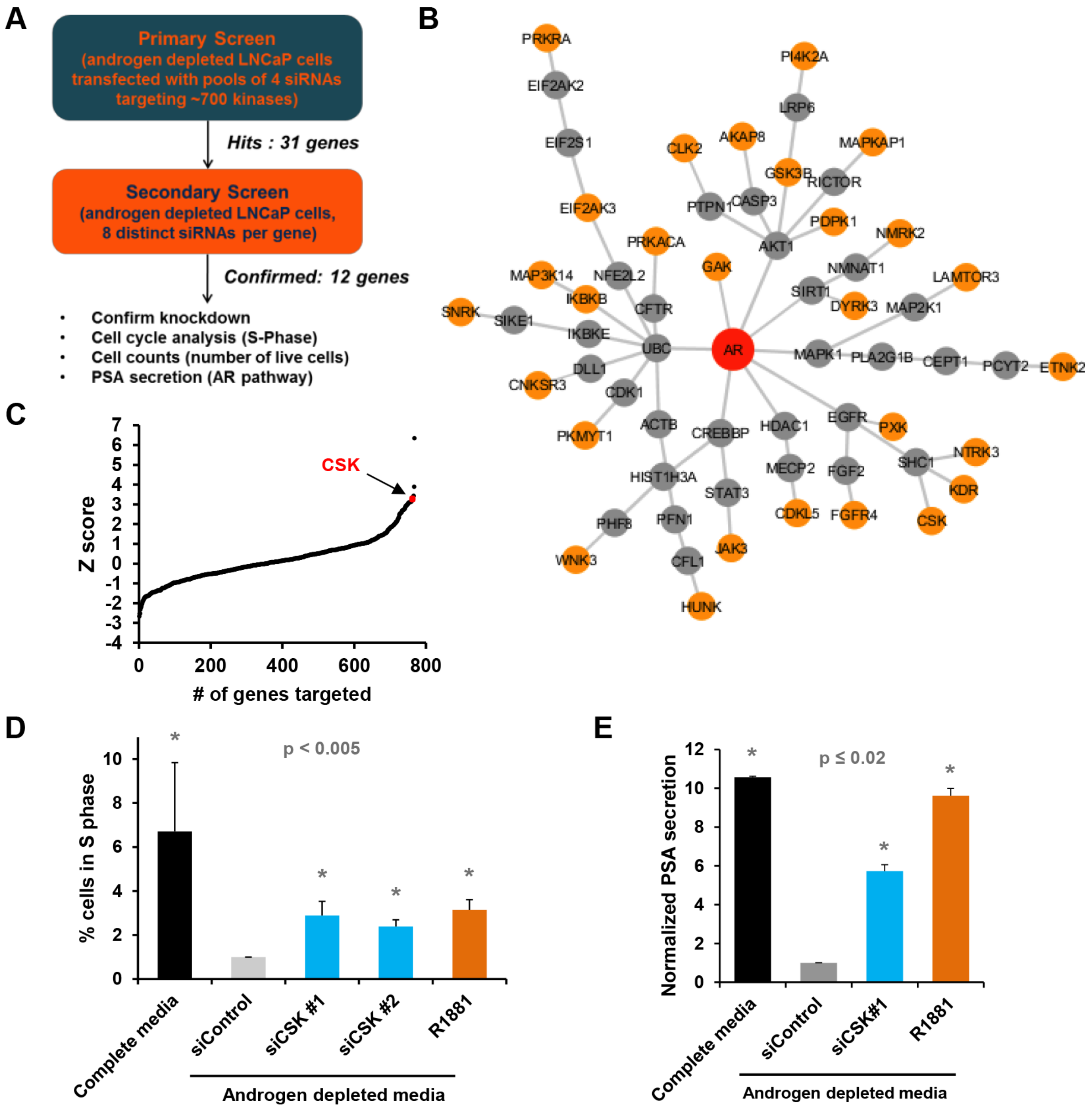

Supplementary Figure S1: SiRNA screening setup and validation. A. Flowchart of the kinome siRNA screen in LNCaP cells. **B.** Network analysis of the primary screening hits demonstrates close clustering around the androgen receptor as a central node (p = 0.045). **C.** CSK was a top hit in the duplicate primary screen and was one of 12 hits validated in subsequent assays. **D.** The effect of knocking down CSK in androgen-depleted LNCaP cells on the proportion of cells in S phase as determined by flow cytometry. Error bars represent standard deviations of 2 replicate measurements. Significance of the differences to the siControl sample (grey bar) was assessed by calculating p values using an unpaired t-test, two-tailed distribution, assuming equal variance. Asterisks denote significant differences. **E.** Effect of CSK knockdown on PSA secretion as determined by ELISA. Error bars represent standard deviations of 2 replicates. Significance of the differences to the siControl sample (grey bar) was assessed by calculating p values using an unpaired t-test, two-tailed distribution, assuming equal variance. Asterisks denote significant differences.

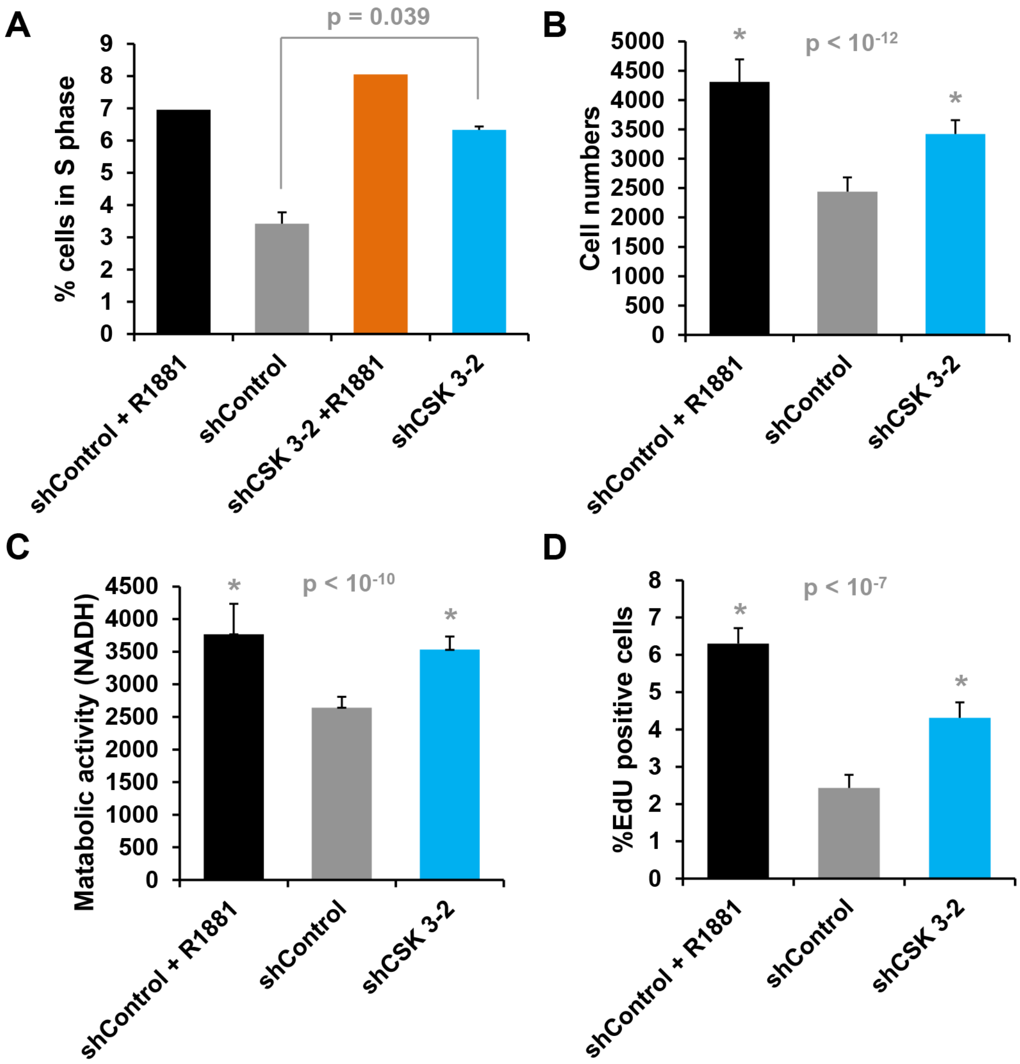

Supplementary Figure S2: Characterization of the shCSK 3–2 cell line. LNCaP cells in which CSK was stably knocked down (shCSK3–2) or shControl cells were maintained in androgen depleted media with or without R1881 supplementation as indicated followed by the determination of **A.** the percentage of cells in S phase, **B.** cell numbers, **C.** metabolic activity (MTS assay) as a measure of proliferation, **D.** EdU incorporation. Error bars represent standard deviations of 2 (A), 16 (B), 22 (C) and 10 (D) replicate measurements. Significance of the differences to the siControl sample (grey bar) was assessed by calculating *p* values using an unpaired *t*-test, two-tailed distribution, assuming equal variance. Asterisks denote significant differences.

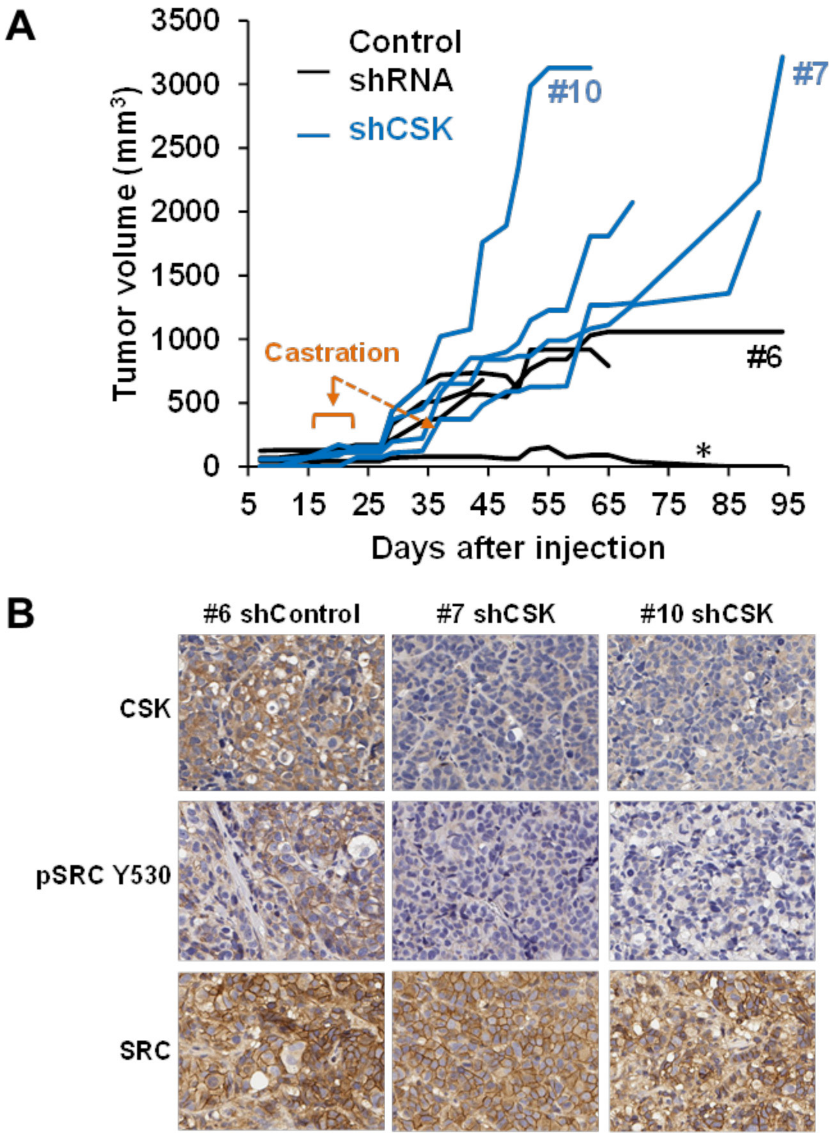

Supplementary Figure S3: CSK knockdown confers castration resistance to LAPC4 cells *in vivo*. A. LAPC4 cells in which CSK was stably knocked down or shControl cells (see Figure 2C) were injected into SCID mice, and tumor growth was monitored. When tumors reached a volume of 100 – 200 mm^3 (typically between days 15 and 23), mice were castrated, and tumor growth was followed for the indicated periods. One mouse injected with shCSK cells which grew slowly was castrated only on day 37 (stippled arrow). One mouse injected with shControl cells did not form tumors and was thus not castrated (indicated by asterisk). **B.** Tumors were excised from mice, fixed and processed for immunohistochemistry staining with antibodies against CSK, SRC, and pSRC Y530.

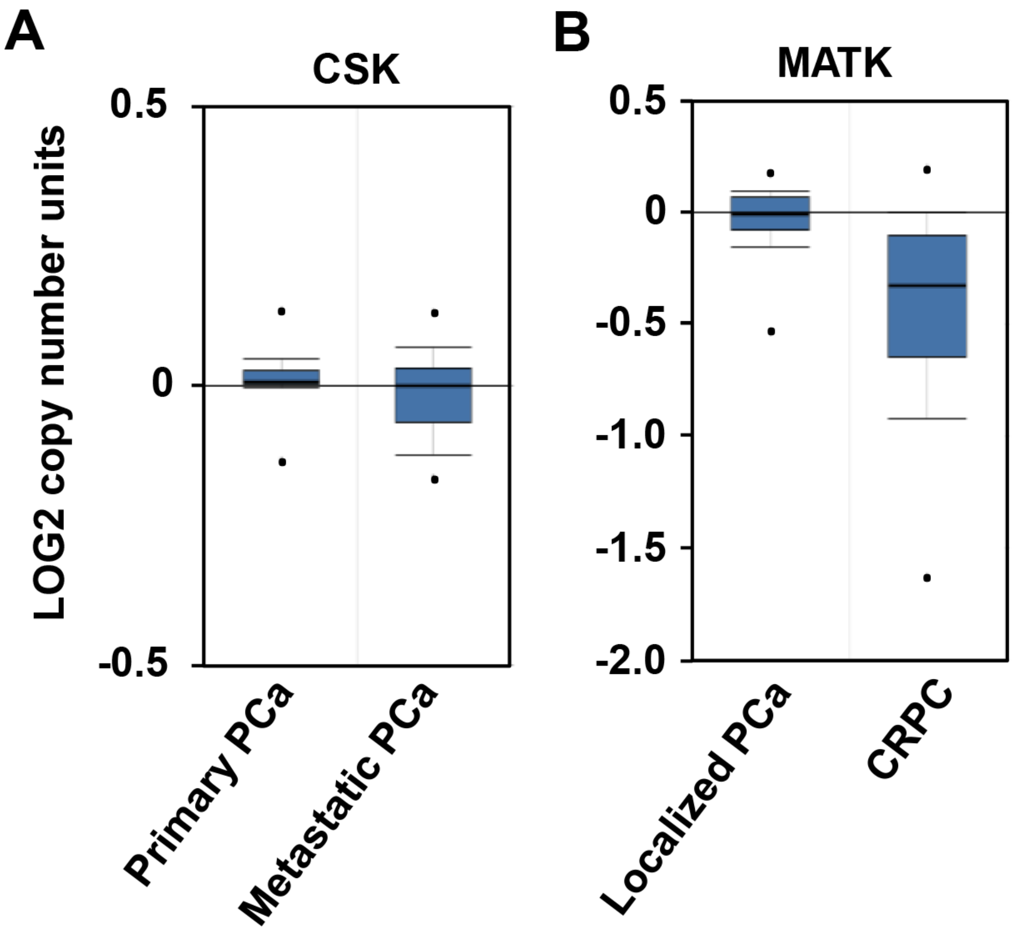

Supplementary Figure S4: Copy number changes of CSK and MATK in human prostate cancers. CSK **A.** and MTAK **B.** gene copy number data from Taylor et al. ((A); (Taylor et al., 2010)) and Grasso et al. ((B); (Grasso et al., 2012)) were drawn from the Oncomine database. PCa = prostate cancer.

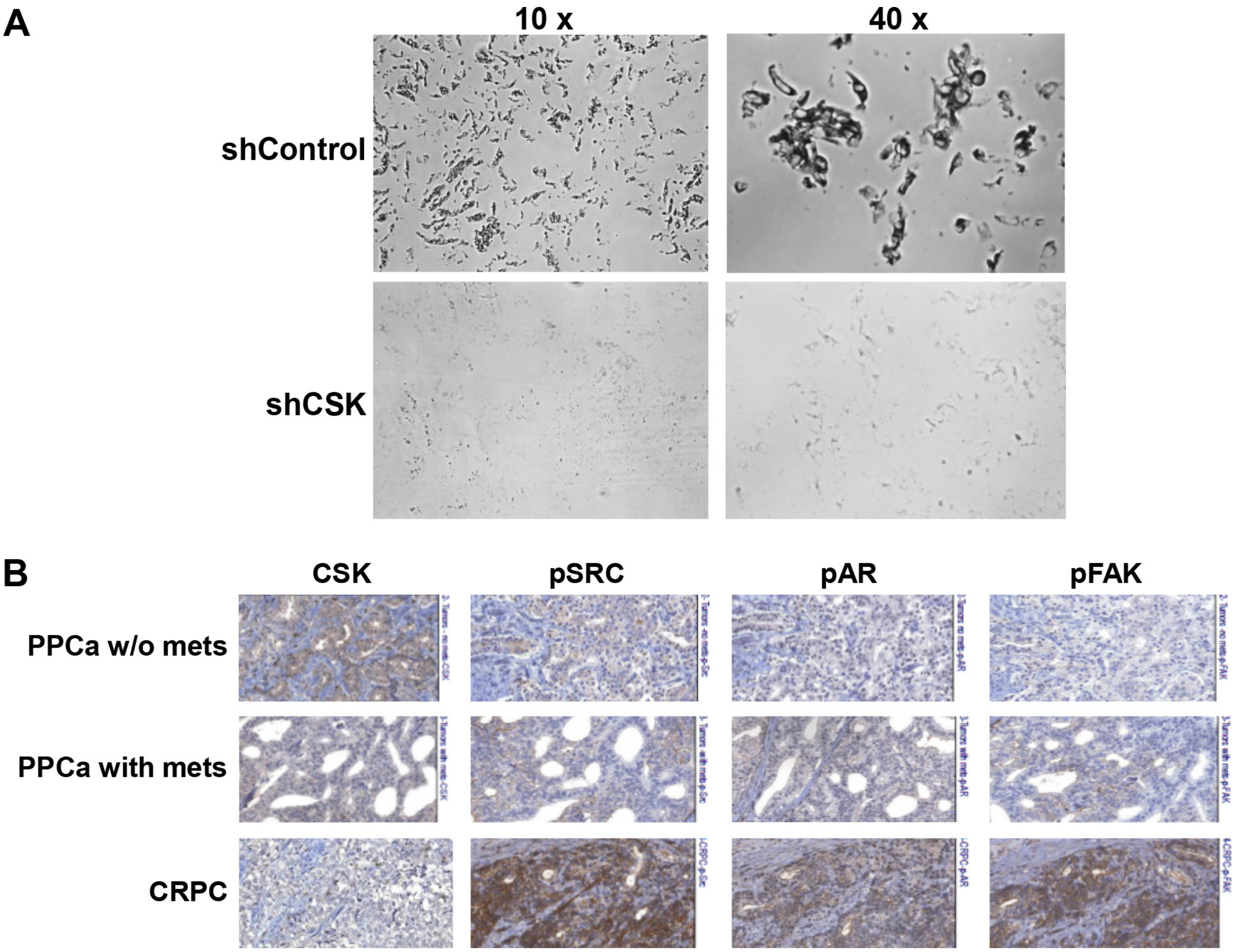

Supplementary Figure S5: Immunohistochemical staining of CSK/SRC pathway components. A. LNCaP cells in which CSK was stably knocked down (shCSK3–2) or shControl cells were embedded in paraffin blocks, sectioned, and stained with an antibody against CSK. The CSK signal is very low in shCSK cells than in shControl cells proving the specificity of the antibody. **B.** Representative examples of the immunohistochemistry results. Tumor microarrays were stained with the indicated antibodies. PPCa = primary prostate cancer; mets = metastases.

Supplementary Table S1: Progression Array - 2011

Groups	# of patients	# of cores	
Tumors with no LN Metastasis	28	56	2 cores each
Tumors with LN Metastasis	20	40	2 cores each
CRPC	28	56	2 cores each
Total	76	152	

REFERENCES

1. Grasso CS, Wu YM, Robinson DR, Cao X, Dhanasekaran SM, Khan AP, Quist MJ, Jing X, Lonigro RJ, Brenner JC, et al. The mutational landscape of lethal castration-resistant prostate cancer. Nature. 2012; 487:239–243.

2. Taylor BS, Schultz N, Hieronymus H, Gopalan A, Xiao Y, Carver BS, Arora VK, Kaushik P, Cerami E, Reva B. Integrative Genomic Profiling of Human Prostate Cancer. Cancer Cell. 2010; 18:11–22.
